# Symptom Management Strategies for Patients Receiving Anaplastic Lymphoma Kinase Inhibitors for Non–Small Cell Lung Cancer

**Published:** 2017-11-01

**Authors:** Jennifer Jacky, Christina Baik

**Affiliations:** Seattle Cancer Care Alliance and Department of Medicine (Medical Oncology Division), University of Washington, Seattle, Washington; and Department of Medicine (Medical Oncology Division), University of Washington School of Medicine, Seattle, Washington

Lung cancer is the second most commonly diagnosed cancer in the United States, with an estimated 222,500 new cases in 2017 (American Cancer Society [ACS], 2017). Non–small cell lung cancer (NSCLC), the most common type (Matsuda & Machii, 2015), has a poor prognosis, with a 5-year survival rate as low as 4% for patients diagnosed with distant metastases (ACS, 2017).

Over the past decade, discoveries of mutations in epidermal growth factor receptor (*EGFR*) and rearrangements in echinoderm microtubule-associated protein-like 4 (*EML4*) anaplastic lymphoma kinase (*ALK*) genes have had a significant impact on NSCLC treatment. Many patients with these mutations benefit from targeted therapies; however, the management of toxicities associated with these agents can be challenging. Here we discuss the distinct adverse effect profile of and symptom management in NSCLC patients treated with ALK inhibitors.

## OVERVIEW OF ALK INHIBITORS

Approximately 3% to 7% of NSCLC patients harbor *ALK* fusions (Kwak et al., 2010). *ALK*-translocated NSCLC is found mostly in younger patients with adenocarcinoma histology and a history of never or light smoking (Inamura et al., 2009; Koivunen et al., 2008; Perner et al., 2008; Shaw et al., 2009; Takeuchi et al., 2008; Wong et al., 2009). Although the frequency of this molecular subtype is low, it is seen relatively often in the clinic, given the high overall incidence of NSCLC. The National Comprehensive Cancer Network (NCCN) recommends that all patients diagnosed with nonsquamous NSCLC or NSCLC not otherwise specified be tested for *ALK* rearrangements so patients with *ALK*-positive tumors can receive targeted treatment (NCCN, 2016).

There are currently four US Food and Drug Administration–approved ALK inhibitors for the treatment of NSCLC: crizotinib (Xalkori), ceritinib (Zykadia), alectinib (Alecensa), and brigatinib (Alunbrig). Crizotinib and alectinib are approved for first-line treatment of patients with locally advanced or metastatic *ALK*-positive NSCLC (Pfizer, 2014). Although crizotinib has a high response rate (> 60%; Camidge et al., 2012; Kwak et al., 2010; Solomon et al., 2014), patients treated with this agent eventually develop resistance.

Ceritinib and brigatinib are next-generation ALK inhibitors approved for the treatment of patients with ALK-positive NSCLC whose disease has progressed on or who are intolerant to crizotinib (Genentech USA, Inc, 2015; Novartis Pharmaceuticals Corporation, 2014). Overall response rates in clinical trials among patients who previously received ALK inhibitors (mostly crizotinib) were 56% and 50% with ceritinib and alectinib, respectively (Kim et al., 2016; Ou et al., 2016). Lorlatinib is a third-generation ALK inhibitor that is currently being evaluated in clinical trials. 

Patients can remain on ALK inhibitor treatment for long periods. In a phase III study of ALK inhibitor–naive patients treated with crizotinib, median progression-free survival (PFS) was 7.7 months (Shaw et al., 2013). In a phase I study of patients treated with ceritinib whose disease progressed on chemotherapy or crizotinib, median PFS was 8.9 months (Shaw et al., 2014). Furthermore, median PFS among crizotinib-naive patients who received ceritinib was 18.4 months (Kim et al., 2016). Thus, it is critical to manage adverse effects to support long-term dosing.

**Managing Distinct Adverse Events**

Targeted therapies for metastatic NSCLC are associated with a distinct set of adverse effects that differ from those seen with chemotherapies. Optimal symptom or adverse effect management should be patient-centered and sensitive to treatment issues arising in the palliative setting and involve both pharmacologic and nonpharmacologic strategies. As such, recommendations will vary from patient to patient. Close collaboration among nurse practitioners, physician assistants, oncologists, pharmacists, and nurses is key to providing optimal care that allows patients to remain on therapy that is both tolerable and effective in controlling cancer growth.

Our institution has been actively involved in clinical trials of ALK inhibitors, and we have gained extensive experience in managing patients on these agents. We believe other clinicians may benefit from our group’s experience in managing supportive care within the framework of NCCN Guidelines (NCCN, 2015a, 2015b).

## CASE STUDY 1

**Figure 1:**
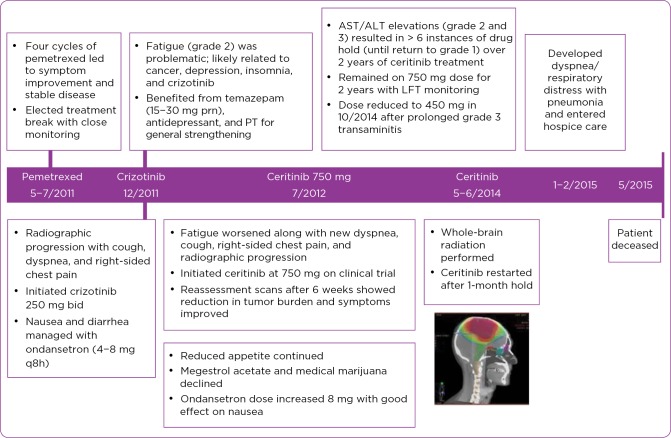
Treatment timeline for the patient in Case Study 1: 79-year-old woman, never smoker. prn = as needed; PT = physical therapy; AST = aspartate transaminase; ALT = alanine transaminase; LFT = liver function test; bid = twice daily.

Ms. P, a 79-year-old female who never smoked, presented in April 2011 with *EML4-ALK* translocation–positive metastatic adenocarcinoma of the lung (see treatment timeline, Figure 1). She had a right-sided pleural effusion, right lower-lobe mass, bone disease, and an Eastern Cooperative Oncology Group Performance Status (ECOG PS) of 2.

**Figure 2:**
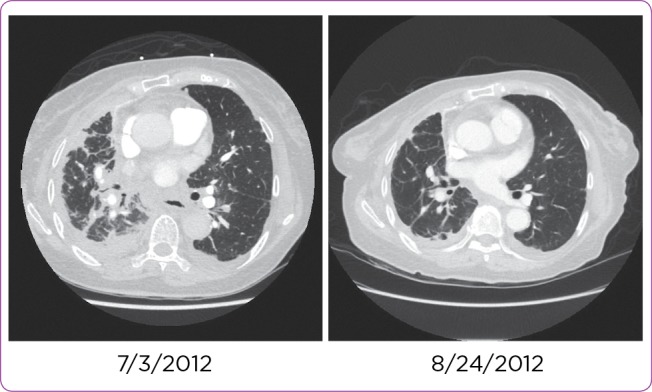
CT scans of the patient in Case Study 1 before targeted therapy (left) and showingresponse within 1 week of starting ceritinib (right).

She was first treated with pemetrexed (Alimta) monotherapy. After four cycles of treatment, her symptoms improved, and reassessment scans demonstrated stable disease. She tolerated therapy but elected a treatment break with close monitoring. By December 2011, there was imaging evidence of tumor progression, and she reported increased cough, shortness of breath, and right-sided tumor-associated chest pain. She started crizotinib at 250 mg twice daily, and within 1 week, symptoms associated with disease progression improved (Figure 2). She also experienced nausea and diarrhea, which were managed with ondansetron.

**Fatigue**

A few months after starting crizotinib, Ms. P reported fatigue, which was attributed to multiple factors (cancer, insomnia, depression, and crizotinib). She was treated with low-dose trazodone and an antidepressant, as some of her insomnia was related to depression and anxiety. She reported an improvement in sleep and reduced fatigue and was referred to physical therapy for general strengthening. Despite having residual fatigue, she was able to continue activities with her family and enjoyed some travel.

After Ms. P reported gait instability, a brain magnetic resonance imaging (MRI) scan showed small intracerebral lesions that did not appear to be responsible for her symptoms. She declined radiosurgery and continued with surveillance. Her gait problems and central nervous system lesions did not progress. Over time, her fatigue worsened; she developed new shortness of breath and right-sided chest pain and cough, and imaging showed disease progression after 6 months of treatment with crizotinib (at 250 mg twice daily). She then enrolled in a clinical trial and was treated with ceritinib at 750 mg daily.

**Anorexia**

After 4 to 5 weeks of starting ceritinib at 750 mg daily, Ms. P reported reduced appetite, which was attributed to ceritinib. Reassessment scans performed after 6 weeks of treatment demonstrated reduced tumor burden in her chest and brain. Disease-associated chest pain, cough, and dyspnea improved; however, her reduced appetite continued, and she was referred to a nutritionist. Megestrol acetate and medical marijuana were discussed as treatment options, but Ms. P declined, and her weight remained within 5% of baseline. Nausea was contributing to her reduced appetite, and the dose of ondansetron was increased, with good effect.

**End of Life**

Ms. P continued ceritinib for 2 years, with several drug interruptions associated with transaminitis. She elected to hold ceritinib and undergo whole-brain radiation after progression of metastatic disease in the brain in May 2014. She restarted ceritinib at 750 mg daily after 1 month. She subsequently experienced transaminitis at levels warranting dose reduction to 600 mg daily and then 450 mg daily. She had increased fatigue after brain radiation and had gait disturbances, although her disease was stable. In January 2015, she developed dyspnea and respiratory distress from pneumonia. She required hospitalization and was discharged with hospice care because of declining performance status in February. She died at home in May 2015.

## CASE STUDY 2

**Figure 3:**
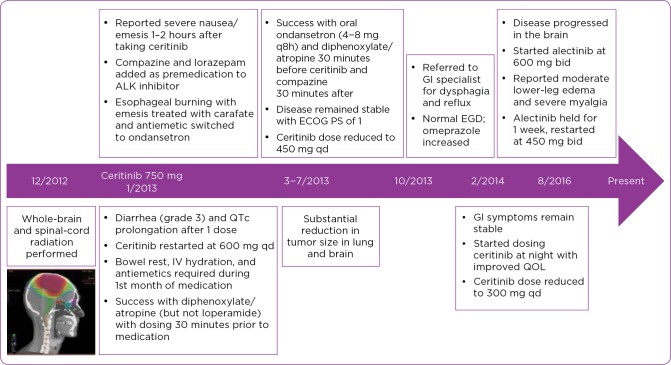
Treatment timeline for the patient in Case Study 2: 45-year-old woman, never smoker presenting with multiple metastatic sites. qd = once daily; IV = intravenous; ECOG PS = Eastern Cooperative Oncology Group performance status; GI = gastrointestinal; EGD = esophagogastroduodenoscopy; QOL = quality of life.

Ms. W, a 45-year-old woman who never smoked, presented in December 2012 with *ALK* translocation–positive adenocarcinoma of the lung (Figure 3). She had a left lower-lobe mass, pleural effusion, bone lesions at T12 and L3, and multiple brain metastases (> 50), with an ECOG PS of 2. She completed whole-brain and palliative radiation from T12 to L3 in January 2013.

**Diarrhea**

Ms. W started a clinical trial with ceritinib at 750 mg daily in January 2013. After one dose, she developed watery diarrhea and a prolonged QTc interval. Ceritinib was reduced to 600 mg daily. During the first month of treatment, she required bowel rest, intravenous hydration, and antiemetics. She tried loperamide, without success, but found diphenoxylate/atropine dosed 30 minutes before treatment to be helpful.

**Nausea**

Ms. W also reported severe nausea 1 to 2 hours after taking ceritinib. She met with a nutritionist about her weight loss and was instructed to eat small meals and educated about a constipating diet. For nausea, prochlorperazine and lorazepam were added as premedication 30 minutes prior to ceritinib. One month into therapy, Ms. W reported esophageal burning with nausea and was prescribed sucralfate, which was then switched to ondansetron. Fearing constipation, she stopped using diphenoxylate/atropine and had diarrhea as a result. Upon restarting diphenoxylate/atropine, her diarrhea was better controlled.

**Symptom Management Over 3 Years**

After 2 months of treatment, there was significant tumor reduction in Ms. W’s lungs and brain. She continued to manage her symptoms for 3 months by taking oral ondansetron and diphenoxylate/atropine 30 minutes before ceritinib and prochlorperazine 30 minutes after ceritinib. She was reticent to take ondansetron and diphenoxylate/atropine later in the day and was continually encouraged to use them as needed. Her electrolytes remained stable despite ongoing diarrhea and occasional nausea. She took ondansetron and diphenoxylate/atropine up to three times daily and dosed prochlorperazine for refractory nausea, with satisfactory control. Her disease remained stable, with an ECOG PS of 1. Despite receiving best supportive care, the patient desired a dose reduction to help mitigate the gastrointestinal adverse effects. Her dose was reduced to 450 mg, with partial improvement of her symptoms.

In October 2013, Ms. W was referred to a gastrointestinal specialist for dysphagia and reflux because of concerns that strictures from radiation were contributing to her symptoms. She underwent esophagogastroduodenoscopy, which was normal, and omeprazole was increased.

In December 2014, Ms. W required a dose reduction to ceritinib at 300 mg daily because of transaminitis. Her gastrointestinal symptoms remained stable and reasonably managed on ceritinib at this dosage.

**Myalgia and Edema**

In August 2016, Ms. W was found to have disease progression in the brain, although her disease was controlled systemically. She opted to start alectinib at 600 mg twice daily; within 1 week, she developed moderate lower-leg edema and severe diffuse myalgia and joint pain. She was treated symptomatically with nonsteroidal anti-inflammatory drugs (NSAIDs); however, this approach was not effective. Blood work showed that her creatinine kinase (CK) levels were elevated. Her symptoms and CK levels improved after holding alectinib treatment for a week; she then restarted alectinib at a reduced dose of 450 mg twice daily. As of the writing of this manuscript, Ms. W was still being treated and doing well at the reduced dose, with some peripheral edema but no significant myalgia or joint pain.

## DISCUSSION

**Fatigue**

Patients on targeted agents commonly report fatigue, which is assessed using a visual analog scale and graded so interventions can be evaluated. When blood work or imaging does not reveal a reversible cause, such as hypothyroidism, anemia, or progressive disease, we screen the patient for insomnia, sleep apnea, and depression and look closely for issues related to polypharmacy. As in the case of Ms. P (Case Study 1), adequate treatment of depression and insomnia may often help with fatigue. For persistent fatigue that is refractory to these measures, we may try stimulants as recommended by the NCCN (NCCN, 2015b). We do not routinely prescribe modafinil or steroids for this purpose.

Referrals to physical therapy can be helpful if the patient has specific deficits or needs guidance in increasing activity. We encourage regular daily activities and exercise as able, particularly those that patients enjoy. However, even with these interventions, some fatigue may persist. It is important to discuss this with patients to manage their expectations.

**Anorexia**

Reduced appetite and weight loss are common symptoms reported by patients with cancer, including those treated with targeted agents. Potential contributors to reduced appetite include pain, nausea, constipation, depression, and mucositis or taste changes. Referral to a nutritionist may be offered.

We first recommend scheduling routine, small, calorie-dense meals to ensure adequate intake and experimenting with different textures and tastes. We generally reserve starting megestrol acetate, olanzapine (Zyprexa), or mirtazapine until the patient has demonstrated weight loss, and we discuss risks and benefits. Medical marijuana is available in the state of Washington and has been helpful for many patients, although the evidence remains mixed on its overall efficacy (Davis, 2016). Patients should be monitored for QTc prolongation with the combination of olanzapine and megestrol acetate.

**Diarrhea**

For diarrhea, we start with loperamide and then diphenoxylate/atropine. We ask patients to monitor the timing of diarrhea and time medications accordingly. Tincture of opium can be used for refractory cases. Some patients require two antidiarrheal medications prior to dosing ALK inhibitors, with additional medication a few hours later. Patients are counseled that constipation can occur with chronic antidiarrheal medication. We monitor electrolytes often and replace fluids as necessary for patients reporting more than three episodes of diarrhea daily. Patients with persistent diarrhea despite optimal management require dose reduction.

**Nausea and Vomiting**

ALK inhibitors have moderate to high emetogenic potential; patients should have antiemetic medications for home use (NCCN, 2015a). First, we evaluate the medical causes of emesis, such as electrolyte abnormalities, medical comorbidities, other medications, and metastatic brain disease. We generally find benefit from ondansetron, as it can help with both nausea and diarrhea. Prochlorperazine or lorazepam may also help patients with refractory symptoms. We reserve starting metoclopramide, as the promotility effect of this medication theoretically may exacerbate diarrhea. Olanzapine is now recommended by the NCCN and has been shown to be effective for patients receiving highly emetogenic chemotherapy (Navari et al., 2016).

Ondansetron, prochlorperazine, and olanzapine can cause prolonged QTc interval. Both crizotinib and ceritinib are associated with QTc prolongation. Concomitant use of supportive medications associated with QTc prolongation risk should be monitored carefully. We recommend a baseline electrocardiogram, which should be repeated at the medication’s steady state. Routine monitoring should also include electrolytes and frequent review of medication lists.

Dosing antiemetic medications 30 to 40 minutes before cancer treatment may benefit patients who have nausea after taking ALK inhibitors (Schaefer & Baik, 2016). Ms. W (Case Study 2) benefited from close monitoring and encouragement to take medications and manage reflux symptoms. Some ALK inhibitors are approved to be taken with or without food, and, if appropriate, taking them with food may help manage nausea. We may alter the timing of the drug dosing depending on patient comfort. For example, Ms. W found that taking the medication at night was helpful, as it allowed her to better predict and control adverse effects during this time.

We also refer patients to nutritional services for suggestions for a constipating diet and strategies to avoid weight loss. As was the case with Ms. W, some patients report improved quality of life with dosing ALK inhibitors in the evening. Patients with refractory symptoms may benefit from consultation with a gastroenterologist. We also suggest screening patients for psychosocial factors that may contribute to nausea and vomiting.

**Myalgia**

Myalgia is a common side effect of alectinib, with variable degrees of severity. In patients with mild myalgia, supportive therapy with analgesics (NSAIDs, acetaminophen) may be used. Patients should also undergo lab evaluation to monitor electrolytes and CK levels. Treatment hold and dose reduction should be considered in patients with severe pain unresponsive to analgesics or CK levels five times the upper limit of normal or higher.

## CONCLUSIONS

Experience administering ALK inhibitors within our institution may provide valuable guidance for oncology nurses and advanced practice providers who may see these patients infrequently. ALK inhibitors are associated with adverse effects that are distinct from those seen with cytotoxic chemotherapy. Central to managing patients on ALK inhibitors is assessing patient quality of life, given the goals of care in the palliative setting. The Case Study patients have varying presentations and patient characteristics that highlight management strategies for adverse effects with ALK inhibitors. By utilizing strategies concordant with those in the NCCN Guidelines for supportive care, both patients remained on ALK inhibitors for more than 2 years.

**Acknowledgments**

Medical writing and editorial assistance were provided by Stephanie Vadasz, PhD, and Shannon Davis of QXV Communications (Haddam, Connecticut) and funded by Novartis.

## References

[1] American Cancer Society. (2017). Cancer facts & figures.. https://www.cancer.org/content/dam/cancer-org/research/cancer-facts-and-statistics/annual-cancer-facts-and-figures/2017/cancer-facts-and-figures-2017.pdf.

[2] Camidge D Ross, Bang Yung-Jue, Kwak Eunice L, Iafrate A John, Varella-Garcia Marileila, Fox Stephen B, Riely Gregory J, Solomon Benjamin, Ou Sai-Hong I, Kim Dong-Wan, Salgia Ravi, Fidias Panagiotis, Engelman Jeffrey A, Gandhi Leena, Jänne Pasi A, Costa Daniel B, Shapiro Geoffrey I, Lorusso Patricia, Ruffner Katherine, Stephenson Patricia, Tang Yiyun, Wilner Keith, Clark Jeffrey W, Shaw Alice T (2012). Activity and safety of crizotinib in patients with ALK-positive non-small-cell lung cancer: updated results from a phase 1 study.. *The Lancet. Oncology*.

[3] Davis Mellar P (2016). Cannabinoids for Symptom Management and Cancer Therapy: The Evidence.. *Journal of the National Comprehensive Cancer Network : JNCCN*.

[4] Genentech USA, Inc. (2015). Alecensa (alectinib): Highlights of prescribing information.. http://www.gene.com/download/pdf/alecensa_prescribing.pdf.

[5] Inamura Kentaro, Takeuchi Kengo, Togashi Yuki, Hatano Satoko, Ninomiya Hironori, Motoi Noriko, Mun Ming-yon, Sakao Yukinori, Okumura Sakae, Nakagawa Ken, Soda Manabu, Choi Young Lim, Mano Hiroyuki, Ishikawa Yuichi (2009). EML4-ALK lung cancers are characterized by rare other mutations, a TTF-1 cell lineage, an acinar histology, and young onset.. *Modern pathology : an official journal of the United States and Canadian Academy of Pathology, Inc*.

[6] Kim Dong-Wan, Mehra Ranee, Tan Daniel S W, Felip Enriqueta, Chow Laura Q M, Camidge D Ross, Vansteenkiste Johan, Sharma Sunil, De Pas Tommaso, Riely Gregory J, Solomon Benjamin J, Wolf Jürgen, Thomas Michael, Schuler Martin, Liu Geoffrey, Santoro Armando, Sutradhar Santosh, Li Siyu, Szczudlo Tomasz, Yovine Alejandro, Shaw Alice T (2016). Activity and safety of ceritinib in patients with ALK-rearranged non-small-cell lung cancer (ASCEND-1): updated results from the multicentre, open-label, phase 1 trial.. *The Lancet. Oncology*.

[7] Koivunen Jussi P, Mermel Craig, Zejnullahu Kreshnik, Murphy Carly, Lifshits Eugene, Holmes Alison J, Choi Hwan Geun, Kim Jhingook, Chiang Derek, Thomas Roman, Lee Jinseon, Richards William G, Sugarbaker David J, Ducko Christopher, Lindeman Neal, Marcoux J Paul, Engelman Jeffrey A, Gray Nathanael S, Lee Charles, Meyerson Matthew, Jänne Pasi A (2008). EML4-ALK fusion gene and efficacy of an ALK kinase inhibitor in lung cancer.. *Clinical cancer research : an official journal of the American Association for Cancer Research*.

[8] Kwak Eunice L, Bang Yung-Jue, Camidge D Ross, Shaw Alice T, Solomon Benjamin, Maki Robert G, Ou Sai-Hong I, Dezube Bruce J, Jänne Pasi A, Costa Daniel B, Varella-Garcia Marileila, Kim Woo-Ho, Lynch Thomas J, Fidias Panos, Stubbs Hannah, Engelman Jeffrey A, Sequist Lecia V, Tan WeiWei, Gandhi Leena, Mino-Kenudson Mari, Wei Greg C, Shreeve S Martin, Ratain Mark J, Settleman Jeffrey, Christensen James G, Haber Daniel A, Wilner Keith, Salgia Ravi, Shapiro Geoffrey I, Clark Jeffrey W, Iafrate A John (2010). Anaplastic lymphoma kinase inhibition in non-small-cell lung cancer.. *The New England journal of medicine*.

[9] Matsuda T, Machii R (2015). Morphological distribution of lung cancer from Cancer Incidence in Five Continents Vol. X.. *Japanese Journal of Clinical Oncology*.

[10] National Comprehensive Cancer Network. (2015a). NCCN Clinical Practice Guidelines in Oncology: Antiemesis. v2.2015.. http://www.nccn.org/professionals/physician_gls/pdf/antiemesis.pdf.

[11] National Comprehensive Cancer Network. (2015b). NCCN Clinical Practice Guidelines in Oncology: Cancer Related Fatigue. v2.2015.. http://www.nccn.org/professionals/physician_gls/pdf/fatigue.pdf.

[12] National Comprehensive Cancer Network. (2016). NCCN Clinical Practice Guidelines in Oncology: Non-Small Cell Lung Cancer. v4.2016.. http://www.nccn.org/professionals/physician_gls/pdf/nscl.pdf.

[13] Navari R M, Qin R, Ruddy K J, Liu H, Powell S F, Bajaj M, Loprinzi C L (2016). Olanzapine for the prevention of chemotherapy-induced nausea and vomiting.. *New England Journal of Medicine*.

[14] Novartis Pharmaceuticals Corporation. (2014). Zykadia (ceritinib): Highlights of prescribing information.. https://www.hcp.novartis.com/products/zykadia/#important-safety-info.

[15] Ou Sai-Hong Ignatius, Ahn Jin Seok, De Petris Luigi, Govindan Ramaswamy, Yang James Chih-Hsin, Hughes Brett, Lena Hervé, Moro-Sibilot Denis, Bearz Alessandra, Ramirez Santiago Viteri, Mekhail Tarek, Spira Alexander, Bordogna Walter, Balas Bogdana, Morcos Peter N, Monnet Annabelle, Zeaiter Ali, Kim Dong-Wan (2016). Alectinib in Crizotinib-Refractory ALK-Rearranged Non-Small-Cell Lung Cancer: A Phase II Global Study.. *Journal of clinical oncology : official journal of the American Society of Clinical Oncology*.

[16] Perner Sven, Wagner Patrick L, Demichelis Francesca, Mehra Rohit, Lafargue Christopher J, Moss Benjamin J, Arbogast Stefanie, Soltermann Alex, Weder Walter, Giordano Thomas J, Beer David G, Rickman David S, Chinnaiyan Arul M, Moch Holger, Rubin Mark A (2008). EML4-ALK fusion lung cancer: a rare acquired event.. *Neoplasia (New York, N.Y.)*.

[17] Pfizer, Inc. (2014). Xalkori (crizotinib): Highlights of prescribing information.. http://labeling.pfizer.com/showlabeling.aspx?id=676.

[18] Schaefer Eric S, Baik Christina (2016). Proactive management strategies for potential gastrointestinal adverse reactions with ceritinib in patients with advanced ALK-positive non-small-cell lung cancer.. *Cancer management and research*.

[19] Shaw Alice T, Kim Dong-Wan, Mehra Ranee, Tan Daniel S W, Felip Enriqueta, Chow Laura Q M, Camidge D Ross, Vansteenkiste Johan, Sharma Sunil, De Pas Tommaso, Riely Gregory J, Solomon Benjamin J, Wolf Juergen, Thomas Michael, Schuler Martin, Liu Geoffrey, Santoro Armando, Lau Yvonne Y, Goldwasser Meredith, Boral Anthony L, Engelman Jeffrey A (2014). Ceritinib in ALK-rearranged non-small-cell lung cancer.. *The New England journal of medicine*.

[20] Shaw Alice T, Kim Dong-Wan, Nakagawa Kazuhiko, Seto Takashi, Crinó Lucio, Ahn Myung-Ju, De Pas Tommaso, Besse Benjamin, Solomon Benjamin J, Blackhall Fiona, Wu Yi-Long, Thomas Michael, O’Byrne Kenneth J, Moro-Sibilot Denis, Camidge D Ross, Mok Tony, Hirsh Vera, Riely Gregory J, Iyer Shrividya, Tassell Vanessa, Polli Anna, Wilner Keith D, Jänne Pasi A (2013). Crizotinib versus chemotherapy in advanced ALK-positive lung cancer.. *The New England journal of medicine*.

[21] Shaw Alice T, Yeap Beow Y, Mino-Kenudson Mari, Digumarthy Subba R, Costa Daniel B, Heist Rebecca S, Solomon Benjamin, Stubbs Hannah, Admane Sonal, McDermott Ultan, Settleman Jeffrey, Kobayashi Susumu, Mark Eugene J, Rodig Scott J, Chirieac Lucian R, Kwak Eunice L, Lynch Thomas J, Iafrate A John (2009). Clinical features and outcome of patients with non-small-cell lung cancer who harbor EML4-ALK.. *Journal of clinical oncology : official journal of the American Society of Clinical Oncology*.

[22] Solomon Benjamin J, Mok Tony, Kim Dong-Wan, Wu Yi-Long, Nakagawa Kazuhiko, Mekhail Tarek, Felip Enriqueta, Cappuzzo Federico, Paolini Jolanda, Usari Tiziana, Iyer Shrividya, Reisman Arlene, Wilner Keith D, Tursi Jennifer, Blackhall Fiona (2014). First-line crizotinib versus chemotherapy in ALK-positive lung cancer.. *The New England journal of medicine*.

[23] Takeuchi Kengo, Choi Young Lim, Soda Manabu, Inamura Kentaro, Togashi Yuki, Hatano Satoko, Enomoto Munehiro, Takada Shuji, Yamashita Yoshihiro, Satoh Yukitoshi, Okumura Sakae, Nakagawa Ken, Ishikawa Yuichi, Mano Hiroyuki (2008). Multiplex reverse transcription-PCR screening for EML4-ALK fusion transcripts.. *Clinical cancer research : an official journal of the American Association for Cancer Research*.

[24] Wong Daisy Wing-Sze, Leung Elaine Lai-Han, So Kimpton Kam-Ting, Tam Issan Yee-San, Sihoe Alan Dart-Loon, Cheng Lik-Cheung, Ho Kwok-Keung, Au Joseph Siu-Kie, Chung Lap-Ping, Pik Wong Maria (2009). The EML4-ALK fusion gene is involved in various histologic types of lung cancers from nonsmokers with wild-type EGFR and KRAS.. *Cancer*.

